# Police Response to Domestic and Family Violence Incidents Where the Victim Is Willing to Maintain the Relationship with the Perpetrator

**DOI:** 10.1177/10778012251409164

**Published:** 2025-12-23

**Authors:** Masahiro Suzuki, Md Jahirul Islam

**Affiliations:** 1 Department of Criminology, Sociology, and Social Policy, 5156 Loughborough University, Loughborough, UK; 2 607305 Corrections Research, Evaluation and Statistics, Corrective Services New South Wales, Department of Communities and Justice, Sydney, New South Wales, Australia

**Keywords:** domestic and family violence, police responsiveness, police discretion, decision-making process, mixed methods

## Abstract

As domestic and family violence (DFV) grows as a public health concern, effective police responses are critical. Using a mixed-methods approach, we explored the challenges officers face when victims are reluctant to sever ties with perpetrators. Quantitative findings showed officers generally took proactive measures to protect victims, while qualitative data revealed many adopted a victim-centered approach. However, some officers demonstrated limited understanding of the complexities behind victims’ decisions to stay. These findings highlight the need for enhanced training to better equip officers with the knowledge to navigate the multifaceted dynamics of DFV cases.

## Introduction

Domestic and family violence (DFV) is a pervasive and complex societal issue with significant public health implications due to the extensive and multifaceted harm it inflicts on victims. Victims often experience long-term physical, psychological, and social consequences, including injuries, mental health disorders such as post-traumatic stress disorder, and economic and social marginalization (Australian Institute of Health and Welfare, 2024; Ellsberg et al., 2009; Mechanic et al., 2008; Simmons et al., 2018). Thus, the role of police in responding effectively to DFV incidents is critical, not only in ensuring victims’ safety and enforcing the law but also in shaping their access to justice and support services. Research has highlighted that the current police response to DFV is insufficient to address the complexities of DFV. For instance, in Queensland, Australia, where this current research was conducted, the Special Taskforce for Domestic and Family Violence in Queensland (2015) identified significant shortcomings in the police response to DFV. The taskforce reported that existing policing practices often contribute to the revictimization or marginalization of victims, underscoring the urgent need for reform in how law enforcement approaches DFV cases. In response to the taskforce's recommendations, the Queensland Government (2021) committed to enhancing the police response to DFV by implementing comprehensive education and training programs for police officers, as well as developing clear and effective guidelines for handling DFV incidents. As part of these initiatives, the Queensland Police Service (2021) has now elevated DFV to a high-priority issue within their service.

Despite growing recognition of DFV as a serious crime and the widespread implementation of reforms to enhance law enforcement responses, including dedicated DFV units and mandatory charging practices, police officers frequently encounter significant challenges when responding to DFV-related call-outs. One of the most complex and emotionally charged scenarios for police officers arises when victims express a desire to remain in the relationship with the perpetrator. This circumstance complicates the intervention process, as victims’ reluctance to sever ties with their abusers may hinder legal proceedings or safety measures. Indeed, a few studies have highlighted the frustration and ethical dilemmas faced by police officers in such situations, where they often struggle with the decision of whether to pursue legal action or other interventions without the victim's active cooperation (Diemer et al., 2017; Meyer & Reeves, 2021; Myhill, 2019). This predicament is compounded by the fact that law enforcement personnel are tasked with both protecting the victim and respecting their autonomy, creating a difficult balance between enforcing the law and respecting the victims’ decisions.

In this current research, we aim to examine whether and why police officers alter their course of action when responding to DFV incidents where the victims are willing to maintain the relationship with the perpetrators. Although the existing literature has identified a variety of legal and extralegal factors that influence police responses to DFV incidents, to date, no studies have specifically investigated the impact of victims’ reluctance to sever ties with their abusers on police decision-making. Our research is the first to address this critical knowledge gap in the literature. By utilizing a survey questionnaire that presents police officers with different scenarios of DFV incidents (Islam et al., 2024), we seek to explore how officers’ responses may vary depending on the victim's expressed willingness to continue their relationship with the perpetrator. Our research will contribute to a deeper understanding of the complex decision-making processes involved in law enforcement's response to DFV, particularly in situations where the relational dynamics between victims and perpetrators are complicated by the victim's desire to maintain the relationship. Our findings will offer valuable insights into the challenges police officers face in such cases and will inform the development of comprehensive guidelines and training programs designed to enhance the police response to complex DFV incidents.

## Factors Determining Police Response to Domestic and Family Violence

Several factors influence police responses to DFV incidents. This section reviews the relevant literature on this topic. Given the diverse range of determinants, we have adopted the categorization proposed by Dichter et al. (2011), which includes legal factors, extralegal factors, and agency factors.

### Legal Factors

According to Dichter et al. (2011, p. 23), legal factors refer to the “factors that have legal relevance in determining a criminal legal case.” In this regard, research indicated that police are more inclined to arrest perpetrators when there is clear evidence at the scene, such as a witness (Buzawa & Austin, 1993; Myhill, 2019). Notably, multiple studies consistently revealed the increased likelihood of arrest when victim injuries are confirmed (Bachman & Coker, 1995; Mason & Petrie, 2021; Phillips, 2008; Phillips & Gillham, 2010; Phillips & Sobol, 2010; Tatum & Pence, 2015). Moreover, perpetrator substance use has been strongly linked to an increased probability of arrest (Jones & Belknap, 1999; Logan et al., 2006). Finally, if the perpetrator is present when police arrive, the chances of an arrest are significantly heightened (Feder, 1996; Hall, 2005).

### Extralegal Factors

Dichter et al. (2011, p. 23) defined extralegal factors are those that “do not have legal relevance and, therefore, should not factor into arrest decisions,” such as the demographics of the victims and offenders. Some studies found that male DFV perpetrators are more frequently arrested (Dichter et al., 2011; Lally & DeMaris, 2012), while other research suggested that gender has no significant effect on arrest rates (Ho, 2003; Russell, 2018) or even reported a higher likelihood of arrest for female perpetrators (Durfee, 2012; Hamilton & Worthen, 2011). On the other hand, the gender of the victim appears to be an important factor, with cases involving female victims more likely to lead to an arrest (Dawson & Hotton, 2014). Importantly, the intersection of race with arrest rates further complicates the picture. Mason and Petrie (2021) found that incidents involving Black female victims and White male offenders were no more likely to result in an arrest than cases with White female victims. Conversely, research by McCormack and Hirschel (2021) showed that White victims were generally more likely to result in an arrest, regardless of the offender's race.

### Agency Factors

Agency factors to how police departments organize their personnel and allocate resources (Dichter et al., 2011). In this regard, the influence of gender composition within police departments on responses to DFV cases remains inconclusive. Some research has shown that the gender of officers has little impact on how they handle DFV incidents (Stalans & Finn, 2000; Sun, 2007), while others suggested that male officers are more inclined to make arrests (Gracia et al., 2014; Robinson & Chandek, 2000). One significant agency factor is the provision of specialized training. Studies indicated that stereotypes about female DV victims and doubts about their credibility can result in officers responding with less urgency or taking a passive approach (Belknap, 1995; Leung, 2014; Lila et al., 2013). However, officers who receive specific training related to DFV may be more inclined to arrest offenders compared to those without such training (Brennan et al., 2021; Islam & Mazerolle, 2022; Johnson, 2010). Research also suggests that when officers understand that their departments support and reward appropriate actions in DFV cases, particularly making arrests, it increases the likelihood of arrests being made (Johnson, 2010; Johnson & Dai, 2016).

## Current Study

Existing research presents a complex picture of police responses to DFV incidents, suggesting that various factors influence how officers determine their course of action. Our study aims to contribute to this body of literature by specifically investigating how and why police officers may modify their responses when the victim expresses a desire to maintain the relationship with the perpetrator. Previous research has shown a link between the victim's preference for arrest and an increased likelihood of the perpetrator being arrested (Berk & Loseke, 1980; Eigenberg et al., 1996; Phillips & Sobol, 2010). These findings suggest that police officers may encounter challenges in situations where victims are reluctant to sever ties with the perpetrator, particularly since DFV often occurs in private spaces and relies heavily on victim cooperation to move forward legally. By examining not only whether police adjust their actions but also the underlying reasons for these decisions, our research will offer new insights for developing more effective guidelines and training programs for law enforcement officers when responding to DFV cases, particularly in situations where victims are hesitant to end their relationship with the abuser.

## Methodology

### Setting and Sample

The research was conducted within a single state police jurisdiction, which is the third most densely populated state in Australia, with a population of just over five million people (Australian Bureau of Statistics, 2024). Data were collected through an online survey between March and April 2012, targeting a randomly selected, representative group of police officers. The survey aimed to gather information on various aspects of policing DFV, including officers’ perceptions and attitudes toward DFV (Islam et al., 2024). Although the data were collected in 2012, the legal framework and standard police procedures for responding to domestic and family violence in Queensland have remained largely consistent (Queensland Police Service, 2021). While minor procedural updates may have occurred over time, the scenarios used in this study continue to reflect realistic decision-making contexts for police officers. Therefore, the findings remain relevant for understanding policing practices and officer responses to domestic violence.

A total of 1593 officers from different police districts across the state were randomly invited to participate, and 243 officers completed the survey, resulting in a response rate of 16 percent. As part of the study, officers were assigned one of four hypothetical DFV scenarios that depicted a fictional incident and the response of an imaginary officer, Constable Darcy. Officers were then asked how they would respond to a similar situation. A total of 72% of respondents (175 out of 243) answered all the questions related to these scenarios, with the number of responses distributed across them as follows: 47 for Scenario 1, 45 for sScenario 2, 41 for Scenario 3, and 42 for Scenario 4. Chi-square analyses were performed, revealing no significant differences among the scenario groups in terms of gender, age, rank, region, or training background.

### Data Collection Methods

The study utilized an online survey methodology, distributing survey packages to the participating officers. These packages contained an introductory letter, a demographic information sheet, and vignettes accompanied by Likert-type questions (see Appendices A and B). The main tools for data collection included these vignettes and a set of 13 Likert-type questions, along with additional supplementary inquiries. Vignettes were chosen for their effectiveness in illustrating specific scenarios, which can yield more accurate and dependable responses than abstract questions (Alexander & Becker, 1978). Research has demonstrated consistency between hypothetical choices made in vignettes and actual decisions in real-world contexts (Hainmueller et al., 2015). Accordingly, vignettes are widely employed in police research (e.g., Saunders et al., 2016).

Four distinct vignettes were developed to investigate the potential responses of police officers in different DFV contexts. Each vignette shared a similar background while detailing a DFV incident involving a male character, referred to as Mr. Hamilton, and a female character, referred to as Mrs. Hamilton. These narratives depicted a conflict that escalated from a verbal argument to physical violence, resulting in visible injuries, such as a red mark on Mrs. Hamilton's face, and significant emotional distress. The four vignettes were constructed to capture variation in both the severity of violence and the victim's willingness to provide a formal statement. Although not a full factorial design, this structure was chosen to strike a balance between analytic value and practical feasibility, avoiding excessive respondent burden while still enabling examination of key decision-making factors.

Officers were randomly assigned to one of the four vignettes. They were instructed to read their assigned vignette and then answer a 10-point Likert-type question, where 1 indicated “unlikely” and 10 indicated “very likely,” regarding their likely course of action in a similar scenario. Additionally, officers responded to several open-ended questions concerning the perceived severity of the vignette. Finally, they were presented with an alternative version of each scenario and asked if their responses or recommendations would change if Mrs. Hamilton were willing to maintain the relationship with Mr. Hamilton or wished to pursue permanent separation.

### Ethics Approval

The Griffith University Human Research Ethics Committee approved this research. Participants were informed of the study's objectives and the procedures in place to ensure the confidentiality and anonymity of their personal information before completing the survey.

### Analytic Strategy

A mixed methods design was employed to capture the complex responses of police officers to the DFV incidents wherein the victim is willing to maintain the relationship with the perpetrator. The quantitative data were coded and analyzed using SPSS Version 22.0. We calculated the mean ratings from the responses to the Likert-scale questions designed to capture various dimensions of the issue being studied. To identify potential differences in these means across different scenarios, we applied analysis of variance procedures. This method enabled us to assess whether statistically significant differences existed in officers’ mean ratings based on the varying contextual factors presented. Additionally, we conducted Chi-square analyses to explore variations in responses across different scenarios and demographic groups, thereby providing insights into any emerging divergent patterns.

We complemented our quantitative findings with an in-depth analysis of qualitative data. This analysis involved examining the open-ended questions that aimed to clarify officers’ views on DFV incidents where the victim is reluctant to sever the tie with her abuser. For the thorough analysis of police officers’ narratives, we employed thematic analysis. Following the established procedures for thematic analysis outlined by Braun and Clarke (2006), we concentrated on identifying and categorizing recurring patterns of reasoning, which illuminated a detailed overview of the factors influencing officers’ decision-making. NVivo version 14 was used to assist with coding and organizing qualitative data, facilitating a detailed exploration of both commonalities and discrepancies within officers’ narratives.

## Findings

### Quantitative Data

This section presents the results of the quantitative analysis regarding police officers’ likely courses of action in response to the DFV incidents. These findings will serve as the foundation for the subsequent qualitative analysis, which explores whether and how police officers adjust their responses in cases where the victim is willing to maintain the relationship with the perpetrator.

#### Sample Demographics

Table 1 presents the demographic and service-related characteristics of the participating officers. The average age of respondents was 38.7 years (SD = 8.5), with ages ranging from 21 to 60 years. Among the participants, 126 were male and 49 were female. Approximately two-thirds of the officers were married (66.3%), and nearly half had been with the Queensland Police Service for more than 10 years. A notable majority of respondents (73.7%) held the ranks of constable or senior constable, and 85.7% had received formal training for responding to domestic violence incidents.

**Table 1.** Characteristics of the Study Sample (N = 175).

Characteristic	N	%	M (SD)
Age			38.7 (8.5)
Gender			
Male	126	72.0	
Female	49	28.0	
Rank			
Constable	67	38.3	
Senior constable	62	35.4	
Sergeant	44	25.1	
Senior sergeant	2	1.1	
Marital status			
Single	16	9.1	
Married	116	66.3	
De facto	30	17.1	
In a relationship/no cohabiting	7	4.0	
Divorced	2	1.1	
Separated	4	2.3	
Time at police service			
< 1 year	4	2.3	
Between 1 and 2 years	9	5.1	
Between 2 and 5 years	45	25.7	
Between 5 and 10 years	38	21.7	
> 10 years	79	45.1	
*Training received on DV*			
No	25	14.3	
Yes	150	85.7	

*Note.* M (SD) = mean (standard deviation); DV = domestic violence.

#### Officers’ Ratings of the Seriousness of the Scenarios

Regardless of the specific scenario presented (see Table 2), participating officers uniformly recognized the occurrence of DFV, with ratings for each scenario exceeding 9 on a 10-point Likert scale (9.28, 9.07, 9.68, and 9.21). The officers also classified all scenarios as relatively serious, with average ratings surpassing 3 on a 5-point scale (3.09, 3.02, 3.56, and 3.10). Few officers considered any scenario to be “not at all serious” or “extremely serious.”

**Table 2.** Officers’ Mean Ratings of the Seriousness of the Scenarios.

Scenario	Key Differences in the Scenarios	**‘*n*’**	DV occurred?	How serious?
		Total ‘*n*’ = 175	Mean Rating: 1 (Unlikely)-10 (Very Likely)	Mean Rating: 1 (Not at All)-5 (Extremely)
1	Mrs. H … claims that Mr. H slapped her across the face … Mr. H claims that he lost his temper and slapped Mrs. H, but just to get her to stop yelling … Mrs. H is not willing to provide a formal statement	47	9.28	3.09
2	Mrs. H … claims that Mr. H slapped her across the face … Mr. H claims that he lost his temper and slapped Mrs. H, but just to get her to stop yelling … Mrs. H is willing to provide a formal statement	45	9.07	3.02
3	Mrs. H … claims that Mr. H punched her in the face … Mr. H claims that he lost his temper and punched Mrs. Hamilton, but just to get her to stop yelling … Mrs. H is not willing to provide a formal statement	41	9.68	3.56
4	Mrs. H … claims that Mr. H slapped her across the face … Mr. H claims that he lost his temper and pushed Mrs. H, but just to try and stop her from hitting him … Mrs. H is not willing to provide a formal statement	42	9.21	3.10
*P value* across scenarios			n.s.	*p* = .001

*Note.* n.s. = not significant.

#### Officers’ Potential Actions in Each Scenario

Officers were asked to rate their likelihood of taking various actions for each scenario on a 10-point Likert scale, with 1 representing “Unlikely” and 10 indicating “Very likely.” The mean responses for these actions are detailed in Table 3. Significant differences were noted across all four scenarios for two specific actions: initiating an investigation (*p* = .001) and detaining Mr. Hamilton (*p* = .019). Officers showed a greater inclination to detain Mr. Hamilton in Scenario 3, which was rated as the most serious, with mean scores of 6.47, 7.33, 8.41, and 6.86 across the scenarios. In terms of initiating investigations, officers were more likely to do so in Scenarios 2 (6.09) and 3 (5.76) compared to Scenarios 1 (3.98) and 4 (4.12). Conversely, officers were generally unlikely to take no action across all scenarios (mean scores of 3.32, 3.13, 2.39, and 2.26) or to recommend that Mrs. Hamilton seek a private domestic violence order (DVO), with scores of 3.57, 2.93, 2.76, and 2.48. They were also hesitant to suggest filing a cross-application for a DVO against both parties (2.19, 3.09, 2.32, and 3.21) or advising Mrs. Hamilton to relocate (4.28, 3.98, 3.78, and 3.71), although these trends were more pronounced in Scenarios 3 and 4 without reaching statistical significance.

**Table 3.** Officers’ Potential Actions for Each Scenario [Mean Rating: 1 (Unlikely)−10 (Very Likely)].

Possible Actions	Scenario 1	Scenario 2	Scenario 3	Scenario 4	P Value Across Scenarios
No action necessary	3.32	3.13	2.39	2.26	n.s.
Application for a DVO against Mr. Hamilton	7.81	8.17	8.71	7.48	n.s.
Encourage Mrs Hamilton to make a private application for a DVO	3.57	2.93	2.76	2.48	n.s.
Take out DVO applications against both Mr. and Mrs. Hamilton	2.19	3.09	2.32	3.21	n.s.
Advise that Mr. Hamilton could be charged with criminal offenses	7.15	7.48	8.10	6.62	n.s.
Initiate an investigation with a view to charging Mr. Hamilton	3.98	6.09	5.76	4.12	*p* = .001
Refer Mrs. Hamilton to support services	8.83	8.93	9.05	9.17	n.s.
Conclude that violence would likely to continue in the future	7.49	7.82	7.93	7.86	n.s.
Advise contact police next day in relation to proceeding with criminal charges	6.09	5.67	5.39	6.14	n.s.
Encourage Mrs. Hamilton to move out of the house	4.28	3.98	3.78	3.71	n.s.
Took a statement from Mrs Hamilton to discuss criminal charges the next day	5.51	6.26	5.44	6.33	n.s.
Take Mr. Hamilton into detention for DV	6.47	7.33	8.41	6.86	*p* = .019

*Note.* n.s. = not significant; DVO = domestic violence order; DV = domestic violence.

Across all scenarios, officers consistently demonstrated a willingness to pursue a DVO (mean scores of 7.81, 8.17, 8.71, and 7.48) and inform Mrs. Hamilton of potential criminal charges against Mr. Hamilton (7.15, 7.48, 8.10, and 6.62), with slightly increased inclinations in Scenario 3, related to Mr. Hamilton's actions, though not statistically significant. Officers were generally supportive of encouraging Mrs. Hamilton to consider seeking criminal charges (6.09, 5.67, 5.39, and 6.14) and gathering statements for potential charges (5.51, 6.26, 5.44, and 6.33), but again, these results did not reach statistical significance. Notably, officers showed a strong tendency to refer Mrs. Hamilton to support services across all scenarios (8.83, 8.93, 9.05, and 9.17), although this finding was not statistically significant.

### Qualitative Data

Building on the quantitative findings, we now address the question of whether and why police officers may alter their responses to DFV incidents when the victim is willing to maintain a relationship with the perpetrator. This section presents results from a thematic analysis of police officers’ narratives in response to the survey's open-ended question: “Please explain why your course of action would or would not be different if the victim were willing to maintain the relationship with the perpetrator.”

Before presenting the results of the thematic analysis, it is important to note that the vast majority of officers (n = 160, 91.4%) reported that they would not change their course of action even when the victim expressed a willingness to maintain the relationship with the perpetrator (see Table 4). This trend was consistent across all scenarios (93.6%, 95.6%, 90.2%, and 85.7%, respectively). As such, the analysis below reflects a consolidated response of police officers across all scenarios. Approximately half of the respondents (n = 94, 53.7%) provided voluntary written responses to the open-ended questions.

**Table 4.** The Proportion of Officers Who Agreed/Disagreed with Their Actions Would Differ if the Victim Were Willing to Maintain the Relationship with the Perpetrator.

Scenario	Response	‘n’	Percentage
1	Yes	3	6.4
	No	44	93.6
2	Yes	2	4.4
	No	43	95.6
3	Yes	4	9.8
	No	37	90.2
4	Yes	6	14.3
	No	36	85.7
Total	Yes	15	8.6
	No	160	91.4

Yet, it is essential to highlight that there was considerable variation in the responses to why they would not change their course of action. Moreover, this was the sole open-ended question focusing on potential changes in response related to the victim's willingness to maintain the relationship with the perpetrator. Consequently, our qualitative analysis is primarily descriptive, which positions our research as exploratory in nature.

#### Reasons for Changed Responses When the Victim Wanted to Maintain the Relationship with the Offender

##### Concern for the Victim's Wellbeing

Only a small proportion of respondents (n = 13, 13.8%) indicated that they would alter their course of action if the victim expressed a desire to maintain the relationship with the perpetrator. Yet, it is crucial to note that this change in response would primarily be driven by concern for the victim's wellbeing. These police officers reported that they would offer “relationship advice” aimed at highlighting the potential risks involved in staying in an abusive relationship. Some respondents emphasized the importance of informing the victim about the likelihood of repeated domestic violence incidents. For instance, Officer 69 in Scenario 1 stated, “[I would] explain how many repeat calls for service there are in couples, and from previous experience, it will happen again.” Similarly, Officer 94 in Scenario 1 mentioned providing further advice regarding the cycle of domestic violence:

[I would give] further advice in relation to the cycle of domestic violence—we (coppers) all know that it will happen again … We can’t change that though—only the aggrieved can—and by staying in an abusive relationship they are putting themselves at risk and harming their children mentally (Officer 94 in Scenario 1).

These remarks reflect the officers’ awareness of the cyclical nature of domestic violence and their effort to educate victims about the risks of ongoing abuse. Other respondents indicated that they would directly advise the victim to end the relationship. For example, Officer 220 in Scenario 4 remarked, “Due to the fact that there have been repeated instances of unreported domestic violence, I would recommend the relationship be terminated.” This perspective underscores the officers’ belief that, in cases of chronic abuse, the best course of action for the victim's safety is to leave the relationship.

#### Reasons for Unchanged Responses When the Victim Wanted to Maintain the Relationship with the Offender

The majority of respondents who provided answers to the open-ended question (n = 81, 86.1%) indicated that they would not alter their course of action in the hypothetical DFV incident, even if the victim expressed reluctance to sever ties with the perpetrator. Their decision was grounded in three primary considerations: (1) recognition of the fact that DFV had occurred, (2) concern over the potential for reoccurrence of DFV, and (3) respect for the victim's autonomy, but with no influence on legal enforcement.

##### Recognition of the DFV Occurrence

Eighteen police officers (22.2%) reported that they would not alter their response to the hypothetical DFV incident because they had already detected clear evidence of DFV. For these respondents, the occurrence of DFV was perceived as an undeniable fact within the scenarios, necessitating intervention to prevent further harm to the victim. For example, Officer 93 in Scenario 1 remarked, “DV has occurred with admissions.” This statement highlights the officers’ emphasis on ensuring accountability and protecting the victim once evidence of violence has been confirmed. Further supporting this perspective, Officer 169 in Scenario 3 stated, “[Domestic Violence] Order should still be applied for in this circumstance,” emphasizing the necessity of pursuing a legal response even when challenges arise. Similarly, Officer 178 in Scenario 3 asserted, “She [Mrs. Hamilton] still needs the protection of the DVO [domestic violence order] regardless.” These officers’ responses reflect a professional obligation to act when there is sufficient evidence of DV, prioritizing the immediate safety and welfare of the victim above all else.

##### Concern About the Potential Reoccurrence of DFV

Ten police officers (12.3%) based their decision on the high likelihood of DFV reoccurring if the victim remained in the relationship. Drawing on past experience with DFV cases, Officer 168 in Scenario 3, for instance, asserted that “[Whether the victim is willing to maintain the relationship with the perpetrator] makes no difference. It would be based on [the] likelihood of repeat occurrence, which would obviously be higher if she [Mrs. Hamilton] is staying [in the relationship].” These respondents recognized that the risk of reoccurrence is significantly elevated when victims continue to have contact with their abusers. Indeed, Officer 73 in Scenario 1 reinforced this concern, stating that “with continued contact, an escalation is possible.” Officer 83 in Scenario 1 further elaborated on this point, noting, “In my experience, once DV has started and escalated, it continues.” Their assessments are informed by their extensive field experience, which consistently reveals patterns of repeat abuse in cases where the victim does not sever ties with the abuser. As such, the officers expressed a proactive approach, emphasizing the need for intervention to prevent the victim from being exposed to future harm.

##### Respect for the Victim's Autonomy but with No Influence on Legal Enforcement

Approximately half of the respondents (n = 41, 50.6%) indicated that they respect the victim's decision to remain in the relationship with the abuser, acknowledging the importance of the victim's autonomy. However, they also emphasized that the victim's choice to stay would not affect their response to the hypothetical DFV incident, as their primary obligation is to recognize and respond to the fact that DFV has occurred:

Proceed with the application. Irrelevant whether she [Mrs. Hamilton] stays or not (Officer 81 in Scenario 1).

Mrs. Hamilton is an adult. If she wants to stay it again has no bearing on the evidence available (Officer 147 in Scenario 2).

The presence and actions of police relating to DV are not influenced by the fact that an aggrieved person wishes to remain in the relationship (Officer 167 in Scenario 3).

DV has occurred. It makes no difference if she stays in the relationship or decides to leave (Officer 208 in Scenario 4).

These responses highlight a professional commitment among officers to prioritize the objective evidence of DFV over personal judgments about the victim's relationship choices.

Despite their respect for the victim's autonomy, these officers recognized the potential risks faced by the victim and felt a professional obligation to offer support. For example, Officer 113 in Scenario 1 stated, “I would explain to her that she is within her right to remain with her husband but advise her that there are support agencies to assist her.” This remark indicates that while they refrain from imposing their views on the victim's relationship, they would be willing to provide information about available resources and support. Furthermore, some officers expressed a clear understanding of their role, emphasizing that they do not possess the authority to dictate the victim's choices regarding their relationship:

Most, if not all of them [victims] do. It's not my place to advise someone to leave their partner. I can offer them advice for referrals, and they either take it or not (Officer 109 in Scenario 1).

I provide advice, [and it is] their choice on how they act. She [Mrs. Hamilton] may change her mind later after thinking about the advice given (Officer 155 in Scenario 2).

Advice given would represent the most sensible option. It is up to the parties whether they listen or not (Officer 179 in Scenario 3).

Completing the police application and providing advice/support services is sufficient. I cannot determine that any course of action is better for one person or another, that then becomes a personal opinion (Officer 237 in Scenario 4).

It is essential to note that some police officers (n = 11, 26.8%) expressed nuanced respect for the victim's autonomy to remain in the relationship with the abuser, a perspective that is informed by their pessimistic attitudes regarding the outcomes of DFV incidents. Drawing from their past experiences in handling DFV cases, these officers have come to believe that many victims are likely to return to their abusers, leading to the continued cycle of abuse. This pessimistic outlook is reflected in the remarks of several respondents:

The aggrieved usually goes back to the respondent, so I would expect that to happen regardless of her current plans for the future (Officer 114 in Scenario 1).

Aggrieved persons will often state that the relationship is over however once they calm down (sober up) the relationship continues (Officer 88 in Scenario 1).

Experience finds that most relationships do not end after an incident of domestic violence (Officer 191 in Scenario 3).

These findings suggest that officers’ repeated exposure to recurring patterns of abuse may shape their attitudes toward victim autonomy and influence their responses in DFV cases.

## Discussion

Responding to DFV incidents presents significant challenges for police officers, as each situation is emotionally charged and often highly complex. Our research focused on one such challenging scenario: the victim's reluctance to sever ties with the perpetrator. Specifically, we investigated whether and how police officers modify their response strategies when the victim expresses a desire to maintain the relationship with the perpetrator. The findings from our study revealed that, despite the victim's willingness to continue the relationship and regardless of whether police officers alter their course of action, the vast majority of police officers would still adopt a proactive approach in responding to hypothetical DFV incidents. This proactive stance is primarily driven by a concern for the victim's safety. Most officers reported that they would provide advice on the high likelihood of DFV reoccurrence and inform victims about the availability of support services, such as shelters or counseling. This finding suggests that police are increasingly prioritizing victim protection, regardless of the complexities surrounding the victim's personal decisions. While this finding is encouraging, our research also uncovered a concerning gap in some officers’ understanding of the nuanced challenges that victims face when deciding whether to leave or stay in a relationship with the perpetrator. A small number of officers exhibited pessimistic attitudes toward the potential outcomes of DFV incidents, reflecting a lack of empathy for the economic, emotional, and psychological factors that can influence a victim's decision-making process. Implications of our findings are discussed below.

Despite longstanding criticism of police handling of DFV incidents, our research highlighted two encouraging areas of improvement in police response to DFV incidents. First, there was a strong consensus among officers regarding the seriousness of DFV incidents and the necessity of timely intervention. This finding aligns with the global trends emphasizing the importance of DFV as a critical policing priority (e.g., Robinson et al., 2016). In the context of Australia, this heightened awareness may be partly attributed to recent legislative reforms and the establishment of specialized police units dedicated to DFV cases (Australian Law Reform Commission, 2010). These systemic changes have played a significant role in shaping police practices by providing officers with clearer mandates for intervention. The combination of enhanced training, legislative changes, and the development of specialized units has likely contributed to this heightened awareness and willingness to intervene in DFV cases (Islam & Mazerolle, 2022), even when doing so conflicts with the victim's expressed wishes. This shift indicates that contemporary policing increasingly prioritizes the prevention of further harm in DFV incidents, recognizing the cyclical nature of abuse and the need for timely intervention.

Second, our research highlighted an increasing recognition among police officers of the need to protect victims, even when victims themselves may be reluctant to pursue separation. In line with the findings of Meyer and Reeves (2021), our research has underscored a broad consensus among police officers that protective measures are necessary for DFV incidents, even when these actions may go against the wishes of the victims. This growing acknowledgement may likely reflect the rising influence of a victim-centered approach in policing DFV cases (Birdsall et al., 2017; Hoyle & Sanders, 2000). Such an approach prioritizes the safety and wellbeing of the victim over other considerations, aligning with contemporary best practices in law enforcement. By adopting this approach, police officers have demonstrated an understanding of the complex dynamics of DFV and the necessity of intervention, even when victims may be reluctant or ambivalent about their circumstances. Such a shift in perspective represents a critical step forward in ensuring the long-term protection and support of DFV victims.

While our research revealed several promising findings, it also identified areas for improvement in police responses to DFV. Specifically, some officers demonstrated a limited understanding of the complexities surrounding a victim's decision to stay in or leave an abusive relationship. Contrary to simplistic assumptions, this decision is often burdened by a complex mix of emotional, psychological, and practical difficulties. Research has shown that a victim's choice to stay or leave can be influenced by a wide range of factors (Heron et al., 2022), including awareness of support services (Estrellado & Loh, 2014), the extent of commitment to the relationship (Lacey, 2010), forms of DFV (Ben-Porat & Reshef-Matzpoon, 2023), and the degree of victims’ self-esteem (Kim & Gray, 2008). These factors can intersect in ways that make the decision to leave an abusive partner highly complex and deeply personal. Given these nuances, it is crucial for police officers to cultivate a more comprehensive understanding of the victim's state of mind, particularly regarding their reluctance to sever ties with the perpetrator despite ongoing abuse. Without this understanding, police interventions may be less effective and potentially alienate victims, who might already feel trapped by their circumstances.

These findings underscore the pressing need for enhanced training that equips officers with the knowledge to appreciate the multifaceted nature of a victim's decision-making process in DFV situations. Such training would better prepare law enforcement to provide informed, empathetic, and effective support that addresses both the immediate safety concerns and the broader social and psychological factors at play.

### Limitations and Future Research Directions

This study has several limitations. First, qualitative data were derived from voluntary open-ended responses, meaning that not all participants provided detailed narratives. Therefore, the results may over-represent the views of officers who are more reflective or engaged in the DFV incidents. Second, the sample was drawn solely from a single Australian state, which restricts the generalizability of the results to officers in other regions, where DFV policies and practices may differ. A more comprehensive nationwide survey using face-to-face interviews with randomly selected officers could yield results that are more representative and broadly applicable. Third, while the findings provide valuable insights into police reactions to DFV scenarios, they primarily reflect officers’ tendencies or intentions rather than actual behaviors, as the study relied on hypothetical scenarios. It may be possible that real-world responses could differ significantly from those indicated in vignette-based assessments. Therefore, further research is necessary to explore actual police responses using official data. Fourth, the vignettes focused on incidents involving physical violence and did not consider responses to nonphysical forms of DFV, such as coercive control. Future research should aim to incorporate both violent and nonviolent scenarios to provide a more comprehensive understanding of police responses to DFV. Fifth, although this research suggests that the victim willingness to maintain the relationship with the DFV perpetrator is unlikely to alter the police's course of action, the study did not take into account other circumstantial and contextual factors, such as the presence of children at the scene. Future research should incorporate these contextual variables and explore how different scenarios may shape the relationship between police action and victim willingness to maintain the relationship with the DFV perpetrator.” Sixth, the data were collected more than a decade ago, which may not reflect recent societal and practical changes. Research using more recent data is needed. Finally, the depth of responses varied among participants. While some officers provided detailed explanations, others offered brief or no responses at all. Future studies should delve deeper into the reasons behind police officers’ changing responses to DFV when the victim expresses a desire to maintain the relationship with the perpetrator, as well as examine their attitudes toward DFV more broadly.

## Conclusion

DFV remains a serious and escalating public health issue, causing profound physical, emotional, psychological, and economic harm to victims. As public expectations for improved police responses grow, the need for effective and empathetic intervention has become increasingly urgent. Historically, police were often criticized for inaction or inadequate responses to DFV incidents. However, over time, law enforcement practices have shown notable improvement. Our research supports this shift, finding that officers are increasingly willing to adopt proactive measures in hypothetical DFV scenarios, motivated by a genuine concern for victim safety and wellbeing. This trend is promising and suggests progress in how DFV is approached within policing. Nevertheless, our findings also highlight that some officers demonstrated limited understanding of the complex, multifaceted reasons why victims may choose to remain with their abusers, including emotional attachments and fears of economic insecurity. This lack of insight risks undermining otherwise proactive interventions by failing to fully support victims’ needs. Addressing these gaps through enhanced training is essential to ensure police responses are not only immediate and protective but also informed, empathetic, and sensitive to the realities victims face.

## Notes

1. We have highlighted some words to indicate the differences between each scenario.

## Appendix

### Appendix A: Hypothetical Scenarios Describing Domestic Violence Incidents^1^

Please read the following scenario and respond to the questions about it. Remember, there are no right or wrong answers. Constable Darcy and partner are working a 2:00 pm–10:00 pm shift. They are detailed to attend a domestic violence incident. The informant has telephoned the police to report that their neighbors at 45 Letters Street, the Hamiltons, seem to be having another one of their huge fights, with loud arguing and what sounds like glass being smashed. Information from the Communications Centre operator indicates that there have been no previous calls to this address regarding disturbances and there are no current orders in place. Constable Darcy and partner attend 45 Letters Street. They find that Mrs. Hamilton is visibly distressed and has a large red mark on the side of her face. There is overturned furniture in the house. The officers separate the parties and talk to each individually.

Scenario 1

Mrs. Hamilton confirms that they have just had a fight and claims that Mr. Hamilton **slapped her** across the face. She is adamant that though they yell a lot this is the first time he has hit her. Mr. Hamilton claims that he lost his temper and **slapped Mrs. Hamilton**, but just to **get her to stop yelling**. Constable Darcy canvasses with Mrs. Hamilton the possibility of proceeding with an assault complaint. Mrs. Hamilton is **not willing to provide a formal statement to police regarding the assault**.

Scenario 2

Mrs. Hamilton confirms that they have just had a fight and claims that Mr. Hamilton **slapped her** across the face. She is adamant that though they yell a lot this is the first time he has hit her. Mr. Hamilton claims that he lost his temper and **slapped Mrs. Hamilton**, but just to **get her to stop yelling**. Constable Darcy canvasses with Mrs. Hamilton the possibility of proceeding with an assault complaint. Mrs. Hamilton **is willing to provide a formal statement to police regarding the assault**.

Scenario 3

Mrs. Hamilton confirms that they have just had a fight and claims that Mr. Hamilton **punched her** in the face. She is adamant that though they yell a lot this is the first time he has hit her. Mr. Hamilton claims that he lost his temper and **punched Mrs. Hamilton**, but just to **get her to stop yelling**. Constable Darcy canvasses with Mrs. Hamilton the possibility of proceeding with an assault complaint. Mrs. Hamilton is **not willing to provide a formal statement to police regarding the assault**.

Scenario 4

Mrs. Hamilton confirms that they have just had a fight and claims that Mr. Hamilton **slapped her** across the face. She is adamant that though they yell a lot this is the first time he has hit her. Mr. Hamilton claims that he lost his temper and **pushed Mrs. Hamilton**, but just to **try and stop her from hitting him**. Constable Darcy canvasses with Mrs. Hamilton the possibility of proceeding with an assault complaint. Mrs. Hamilton is **not willing to provide a formal statement to police regarding the assault**.

### Appendix B: Vignette Questions

1. After considering the circumstances, Constable Darcy formed the belief that DV had occurred. If you were Constable Darcy, how likely is it that you would form the same belief?
2. Constable Darcy decided that although DV had occurred, no action was necessary. If you were Constable Darcy, how likely is it that you would do the same?
3. Constable Darcy decided to make an application for a DV protection order against Mr. H. If you were Constable Darcy, how likely is it that you would do the same?
4. Constable Darcy encouraged Mrs. H to make a private application for a DV protection order. If you were Constable Darcy, how likely is it that you would do the same?
5. Constable Darcy decided to take out DVO applications against both Mr. and Mrs. H (a cross-application). If you were Constable Darcy, how likely is it that you would do the same?
6. Constable Darcy advised Mrs. H that Mr. H could be charged with criminal offenses for what he has done. If you were Constable Darcy, how likely is it that you would do the same?
7. Constable Darcy decided to commence an investigation with a view to charging Mr. H with assault occasioning bodily harm. If you were Constable Darcy, how likely is it that you would do the same?
8. Constable Darcy referred Mrs. H to support services. If you were Constable Darcy, how likely is it that you would do the same?
9. Constable Darcy concluded that violence between Mr. and Mrs. H would continue to be a problem in the future. If you were Constable Darcy, how likely is it that you would come to the same conclusion?
10. Constable Darcy told Mrs. H to take some more time to think about it, and to then contact police the next day if she wants further action to be taken in relation to proceeding with criminal charges. If you were Constable Darcy, how likely is it that you would do the same?
11. Constable Darcy encouraged Mrs. H to move out of the house. If you were Constable Darcy, how likely is it that you would do the same?
12. Constable Darcy took a statement from Mrs. H with a view to contacting her the next day to again discuss her options in relation to criminal charges. If you were Constable Darcy, how likely is it that you would do the same?
13. Constable Darcy took Mr. H into detention for DV. If you were Constable Darcy, how likely is it that you would do the same?

## References

[bibr1-10778012251409164] Alexander C. S. Becker H. J. (1978). The use of vignettes in survey research. The Public Opinion Quarterly, 42(1), 93–104. 10.1086/268432

[bibr2-10778012251409164] Australian Bureau of Statistics. (2024). Australian Demographic Statistics. https://www.abs.gov.au/statistics.

[bibr3-10778012251409164] Australian Institute of Health and Welfare. (2024). Family, domestic and sexual violence. https://www.aihw.gov.au/family-domestic-and-sexual-violence/responses-and-outcomes/economic-financial-impacts

[bibr4-10778012251409164] Australian Law Reform Commission. (2010). Family Violence – A National Legal Response. http://www.alrc.gov.au/sites/default/files/pdfs/publications/ALRC114_WholeReport.pdf.

[bibr5-10778012251409164] Bachman R. Coker A. L. (1995). Police involvement in domestic violence: The interactive effects of victim injury, offender’s history of violence, and race. Violence and Victims, 10(2), 91–106. 10.1891/0886-6708.10.2.91 8599601

[bibr6-10778012251409164] Belknap J. (1995). Law enforcement Officers’ attitudes about the appropriate responses to woman battering. International Review of Victimology, 4(1), 47–62. 10.1177/026975809500400104

[bibr7-10778012251409164] Ben-Porat A. Reshef-Matzpoon A. (2023). Stay–leave decision-making among women victims of domestic violence in Israel: Background, interactional, and environmental factors. Journal of Interpersonal Violence, 38(3-4), 3688–3710. 10.1177/08862605221109901 35869596

[bibr8-10778012251409164] Berk S. F. Loseke D. R. (1980). Handling” family violence: Situational determinants of police arrest in domestic disturbances. Law & Society Review, 15(2), 317–346. 10.2307/3053608

[bibr9-10778012251409164] Birdsall N. Kirby S. McManus M. (2017). Police–victim engagement in building a victim empowerment approach to intimate partner violence cases. Police Practice and Research, 18(1), 75–86. 10.1080/15614263.2016.1230061

[bibr10-10778012251409164] Braun V. Clarke V. (2006). Using thematic analysis in psychology. Qualitative Research in Psychology, 3(2), 77–101. 10.1191/1478088706qp063oa

[bibr11-10778012251409164] Brennan I. Myhill A. Tagliaferri G. Tapley J. (2021). Policing a new domestic abuse crime: Effects of force-wide training on arrests for coercive control. Policing and Society, 31(10), 1153–1167. 10.1080/10439463.2020.1862838

[bibr12-10778012251409164] Buzawa E. S. Austin T. (1993). Determining police response to domestic violence victims. American Behavioral Scientist, 36(5), 610–623. 10.1177/0002764293036005006

[bibr13-10778012251409164] Dawson M. Hotton T. (2014). Police charging practices for incidents of intimate partner violence in Canada. Journal of Research in Crime and Delinquency, 51(5), 655–683. 10.1177/0022427814523787

[bibr14-10778012251409164] Dichter M. E. Marcus S. C. Morabito M. S. Rhodes K. V. (2011). Explaining the IPV arrest decision: Incident, agency, and community factors. Criminal Justice Review, 36(1), 22–39. 10.1177/0734016810383333

[bibr15-10778012251409164] Diemer K. Ross S. Humphreys C. Healey L. (2017). A “double edged sword”: Discretion and compulsion in policing domestic violence. Police Practice and Research, 18(4), 339–351. 10.1080/15614263.2016.1230853

[bibr16-10778012251409164] Durfee A. (2012). Situational ambiguity and gendered patterns of arrest for intimate partner violence. Violence Against Women, 18(2), 64–84. 10.1177/1077801212437017 22411299

[bibr17-10778012251409164] Eigenberg H. M. Scarborough K. E. Kappeler V. E. (1996). Contributory factors affecting arrest in domestic and non-domestic assaults. American Journal of Police, 15(4), 27–54. 10.1108/MRR-09-2015-0216

[bibr18-10778012251409164] Ellsberg M. Janse H. A. F. M. Heise L. Watts C. H. García-Moreno C. (2009). Intimate partner violence and women’s physical and mental health in the WHO multi-country study on women’s health and domestic violence: An observational study. Lancet, 371, 1165–1172. 10.1016/s0084-3970(08)79072-8 18395577

[bibr19-10778012251409164] Estrellado A. F. Loh J. M. I. (2014). Factors associated with battered Filipino women’s decision to stay in or leave an abusive relationship. Journal of Interpersonal Violence, 29(4), 575–592. 10.1177/0886260513505709 24212977

[bibr20-10778012251409164] Feder L. (1996). Police handling of domestic calls: The importance of offender’s presence in the arrest decision. Journal of Criminal Justice, 24(6), 481–490. 10.1016/S0047-2352(96)00034-7

[bibr21-10778012251409164] Gracia E. García F. Lila M. (2014). Male police officers’ law enforcement preferences in cases of intimate partner violence versus non-intimate interpersonal violence: Do sexist attitudes and empathy matter? Criminal Justice and Behavior, 41(10), 1195–1213. 10.1177/0093854814541655

[bibr22-10778012251409164] Hainmueller J. Hangartner D. Yamamoto T. (2015). Validating vignette and conjoint survey experiments against real-world behavior. Proceedings of the National Academy of Sciences of the United States of America, 112(8), 2395–2400. 10.1073/pnas.1416587112 PMC4345583 25646415

[bibr23-10778012251409164] Hall D. L. (2005). Domestic violence arrest decision making: The role of suspect availability in the arrest decision. Criminal Justice and Behavior, 32(4), 390–411. 10.1177/0093854805276403

[bibr24-10778012251409164] Hamilton M. Worthen M. G. F. (2011). Sex disparities in arrest outcomes for domestic violence. Journal of Interpersonal Violence, 26(8), 1559–1578. 10.1177/0886260510370592 20724294

[bibr25-10778012251409164] Heron R. L. Eisma M. Browne K. (2022). Why do female domestic violence victims remain in or leave abusive relationships? A qualitative study. Journal of Aggression, Maltreatment and Trauma, 31(5), 677–694. 10.1080/10926771.2021.2019154

[bibr26-10778012251409164] Ho T.-N. (2003). The influence of suspect gender in domestic violence arrests. American Journal of Criminal Justice, 27(2), 183–195. 10.1007/BF02885693

[bibr27-10778012251409164] Hoyle C. Sanders A. (2000). Police response to domestic violence: From victim choice to victim empowerment. British Journal of Criminology, 40(1), 14–36. https://doi.org/10.1093/bjc/40.1.14. https://www.jstor.org/stable/23638528

[bibr28-10778012251409164] Islam M. J. Mazerolle P. (2022). Nexus between police attitudes and responses to domestic and family violence in Australia: Does training matter? Policing and Society, 32(10), 1226–1241. 10.1080/10439463.2022.2029436

[bibr29-10778012251409164] Islam M. J. Suzuki M. Mazerolle P. (2024). Police responses to intimate partner violence incidents involving children: Exploring variations in actions and concerns in an Australian jurisdiction. Child Abuse & Neglect, 147, 106568. 10.1016/j.chiabu.2023.106568 38039762

[bibr30-10778012251409164] Johnson R. R. (2010). Making domestic violence arrests: A test of expectancy theory. Policing (Bradford, England), 33(3), 531–547. 10.1108/13639511011066890

[bibr31-10778012251409164] Johnson R. R. Dai M. (2016). Police enforcement of Domestic Violence laws: Supervisory control or officer prerogatives? Justice Quarterly, 33(2), 185–208. 10.1080/07418825.2014.887762

[bibr32-10778012251409164] Jones D. A. Belknap J. (1999). Police responses to battering in a progressive pro-arrest jurisdiction. Justice Quarterly, 16(2), 249–273. 10.1080/07418829900094131

[bibr33-10778012251409164] Kim J. Gray K. A. (2008). Leave or stay?: Battered women’s decision after intimate partner violence. Journal of Interpersonal Violence, 23(10), 1465–1482. 10.1177/0886260508314307 18309037

[bibr34-10778012251409164] Lacey K. K. (2010). When is it enough for me to leave? Black and Hispanic women’s response to violent relationships. Journal of Family Violence, 25(7), 669–677. 10.1007/s10896-010-9326-4

[bibr35-10778012251409164] Lally W. DeMaris A. (2012). Gender and relational-distance effects in arrests for domestic violence. Crime & Delinquency, 58(1), 103–123. 10.1177/0011128711420102

[bibr36-10778012251409164] Leung L. C. (2014). It’s a matter of trust: Policing domestic violence in Hong Kong. Journal of Interpersonal Violence, 29(1), 82–101. 10.1177/0886260513504645 24176986

[bibr37-10778012251409164] Lila M. Gracia E. García F. (2013). Ambivalent sexism, empathya nd law enforcement attitudes towards partner violence against women among male police offiers. Psychology, Crime and Law, 19(10), 907–919. 10.1080/1068316X.2012.719619

[bibr38-10778012251409164] Logan T. K. Shannon L. Walker R. (2006). Police attitudes toward domestic violence offenders. Journal of Interpersonal Violence, 21(10), 1365–1374. 10.1177/0886260506291653 16940401

[bibr39-10778012251409164] Mason P. B. Petrie M. A. (2021). Exploring officer arrest discretion following state policy changes in intimate partner violence. Violence Against Women, 27(12–13), 2491–2505. 10.1177/1077801220975486 33393868

[bibr40-10778012251409164] McCormack P. D. Hirschel D. (2021). Race and the likelihood of intimate partner violence arrest and dual arrest. Race and Justice, 11(4), 434–453. 10.1177/2153368718802352

[bibr41-10778012251409164] Mechanic M. B. Weaver T. L. Resick P. A. (2008). Mental health consequences of Intimate Partner Abuse: A multidimensional assessment of four different forms of abuse. Violence Against Women, 14(6), 634–654. 10.1177/1077801208319283 PMC2967430 18535306

[bibr42-10778012251409164] Meyer S. Reeves E. (2021). Policies, procedures and risk aversity: Police decision-making in Domestic Violence matters in an Australian jurisdiction. Policing and Society, 31(10), 1168–1182. 10.1080/10439463.2020.1869234

[bibr43-10778012251409164] Myhill A. (2019). Renegotiating domestic violence: Police attitudes and decisions concerning arrest. Policing and Society, 29(1), 52–68. 10.1080/10439463.2017.1356299

[bibr44-10778012251409164] Phillips S. W. (2008). Mandatory arrest: Loosely coupled organisations, situational variables, and the arrest decision. International Journal of Police Science & Management, 10(4), 374–387. 10.1350/ijps.2008.10.4.093

[bibr45-10778012251409164] Phillips S. W. Gillham J. (2010). Policing domestic violence: The significance of charging in the post-arrest decision making of police officers. Partner Abuse, 1(2), 200–219. 10.1891/1946-6560.1.2.200

[bibr46-10778012251409164] Phillips S. W. Sobol J. J. (2010). Twenty years of mandatory arrest: Police decision making in the face of legal requirements. Criminal Justice Policy Review, 21(1), 98–118. 10.1177/0887403408322962

[bibr47-10778012251409164] Queensland Government. (2021). Delivery of Recommendations: Not Now, Not Ever: Putting and End to Domestic and Family Violence in Queensland. https://www.justice.qld.gov.au/initiatives/end-domestic-family-violence/about/not-now-not-ever-report.

[bibr48-10778012251409164] Queensland Police Service. (2021). Domestic & Family Violence Strategy 2021–2023. https://www.qpsdfvinquiry.qld.gov.au/public-hearings/assets/exhibits/week-1/martain-b-attachment-d-dfv-strategy.pdf.

[bibr49-10778012251409164] Robinson A. L. Chandek M. S. (2000). The domestic violence arrest decision: Examining demographic, attiduanal, and situational variables. Crime & Delinquency, 46(1), 18–37. 10.1177/0011128700046001002

[bibr50-10778012251409164] Robinson A. L. Pinchevsky G. M. Guthrie J. A. (2016). Under the radar: Policing non-violent domestic abuse in the US and UK. International Journal of Comparative and Applied Criminal Justice, 40(3), 195–208. 10.1080/01924036.2015.1114001

[bibr51-10778012251409164] Russell B. (2018). Police perceptions in intimate partner violence cases: The influence of gender and sexual orientation. Journal of Crime and Justice, 41(2), 193–205. 10.1080/0735648X.2017.1282378

[bibr52-10778012251409164] Saunders D. G. Prost S. G. Oehme K. (2016). Responses of police officers to cases of officer domestic violence: Effects of demographic and professional factors. Journal of Family Violence, 31(6), 771–784. 10.1007/s10896-016-9822-2

[bibr53-10778012251409164] Simmons S. B. Knight K. E. Menard S. (2018). Long-term consequences of intimate partner abuse on physical health, emotional well-being, and problem behaviors. Journal of Interpersonal Violence, 33(4), 539–570. 10.1177/0886260515609582 26460105

[bibr54-10778012251409164] Special Taskforce on Domestic and Family Violence in Queensland. (2015). Not Now, Not Ever. https://www.justice.qld.gov.au/initiatives/end-domestic-family-violence/about/not-now-not-ever-report.

[bibr55-10778012251409164] Stalans L. J. Finn M. A. (2000). Gender differences in Officers’ perceptions and decisions about domestic violence cases. Women & Criminal Justice, 11(3), 1–24. 10.1300/J012v11n03

[bibr56-10778012251409164] Sun I. Y. (2007). Policing domestic violence: Does officer gender matter? Journal of Criminal Justice, 35(6), 581–595. 10.1016/j.jcrimjus.2007.09.004

[bibr57-10778012251409164] Tatum K. M. Pence R. (2015). Factors that affect the arrest decision in domestic violence cases. Policing (Bradford, England), 38(1), 56–70. 10.1108/PIJPSM-07-2014-0075

